# Height prediction of individuals with osteogenesis imperfecta by machine learning

**DOI:** 10.1186/s13023-024-03433-1

**Published:** 2024-11-09

**Authors:** Hongjiang Yang, Wenbiao Zhu, Bo Li, Hao Wang, Cong Xing, Yang Xiong, Xiuzhi Ren, Guangzhi Ning

**Affiliations:** 1 Department of Orthopedics, International Science and Technology Cooperation Base of Spinal Cord Injury, Tianjin Key Laboratory of Spine and Spinal Cord Injury, Tianjin Medical University General Hospital, Tianjin, China; 2 Tianjin Medical University, Tianjin, China; 3 Department of orthopedics, Children’s Hospital of Soochow University, Suzhou, China

**Keywords:** Osteogenesis imperfecta, Growth curves, Machine learning, Ensemble learning, Deep neural networks, Prediction

## Abstract

**Background:**

Osteogenesis imperfecta (OI) is a genetic disorder characterized by low bone mass, bone fragility and short stature. There is a significant gap in knowledge regarding the growth patterns across different types of OI, and the prediction of height in individuals with OI was not adequately addressed. In this study, we described the growth patterns and predicted the height of individuals with OI employing multiple machine learning (ML) models. Accurate height prediction enables effective monitoring and facilitates the development of personalized intervention plans for managing OI.

**Method:**

This study included cross-sectional data for 323 participants with OI, and the median height Z-score for OI types I, III and IV were − 0.62 (-5.93 ~ 3.24), -3.97 (-10.44 ~ -0.02) and − 1.64 (-6.67 ~ 2.44), respectively. Based on the cross-sectional data of participants, the height curves across different gender and OI types were plotted and compared. Subsequently, feature selection techniques, specifically the filter and wrapper methods, were employed to identify predictive factors for the height of participants. Finally, multiple machine learning (ML) models were constructed for height prediction, and the performance of each model was systematically evaluated.

**Results:**

The analysis of height curves revealed that male with OI are significantly taller than female with OI from the age of 14 (*p* = 0.045), individuals with OI type III are statistically shorter than those with OI types I and IV starting from 3 years old (*p* = 0.006), and those with OI type IV are statistically shorter than those with OI type I from the age of 10 (*p* = 0.028). The application of filter and wrapper methods identified gender (*p* = 0.001), age (*p* < 0.001), Sillence types (*p* = 0.007), weight Z-score (*p* < 0.001) and aBMD Z-score (*p* = 0.021) as significant predictive factors for height. The optimal performance of predictive models was registered by gradient boosting classifier (GB) (bias = 5.783, accuracy = 92.59%, R^2^ = 0.828), random forest (RF) (bias = 6.155, accuracy = 90.12%, R^2^ = 0.788), ensemble machine learning (EML) (bias = 6.250, accuracy = 91.36%, R^2^ = 0.825) and deep neuron networks (DNNs) (bias = 6.223, accuracy = 90.12%, R^2^ = 0.821).

**Conclusion:**

This study analyzed a large cohort of individuals with OI and provided detailed height patterns across different gender and OI types that are crucial for assessing overall growth. Gender, age, Sillence types, weight Z-score and aBMD Z-score were identified as predictive factors for height. The predictive models of GB, RF, EML and DNNs had higher accuracy to evaluate the height of individuals with OI. This study allows guardians and physicians to timely monitor the height parameters, and facilitate the creation of personalized intervention schedules tailored to the needs of individuals with OI.

## Introduction

Osteogenesis imperfecta (OI) is a genetic disorder characterized by low bone mass, bone fragility and short stature [1]. Approximately 90% of OI cases are due to mutations in the gene encoding type I collagen, a major protein component of the extracellular matrix. The remaining 10% involve genes related to the transcription and modification of type I collagen or the regulation of osteoblast differentiation and bone metabolism [2]. The classical Sillence classification, established in 1979 [3], categorized type I collagen-related OI into OI type I (mild), OI type II (perinatally lethal), OI type III (severe) and OI type IV (moderate).

Growth retardation and short stature are prominent in severe forms of OI and are also observed in mild to moderate cases. Researches reported that the length of individuals with OI were generally below compared with normative cases by birth [4, 5]. Other studies demonstrated that final height is obviously restricted, especially in those with OI types III and IV [6–8]. While disease-specific height parameters have been explored in conditions like Down syndrome, Turner syndrome and Achondroplasia [9–11], there are few studies systematically describing the height parameter in the relatively common OI. To date, there was only one study with a large cohort of individuals with OI providing the height parameter, which contributed to the development of OI-specific growth patterns [7].

In the realm of artificial intelligence (AI), which is defined as the simulation of human intelligence by machines, there has been rapid advancement in its application within the medical field [12]. With the applications of AI in medicine setting developing quickly, machine learning (ML) predictive models was widely utilized in automated diagnosis and treatment field. Numerous studies have tried to make early diagnosis for Chronic diseases (CDs) using machine learning [13, 14]. In another study, machine learning was employed to accurately estimate glomerular filtration rate [15]. In addition, we have achieved precise prediction of the length of hospital stay (LOS) by principle component regression [16]. Despite these advancements, the application of AI in the study of OI remains limited.

Here, we reported cross-sectional data from 323 subjects with OI. Firstly, we proposed the height curves in different gender and OI types. Then, the significant predictive factors for the height of individuals with OI were identified. Finally, we construct and compare the performance of multiple machine learning models aimed at predicting height, enhancing our understanding and management of OI through advanced computational techniques.

## Methods

**Fig. 1:**
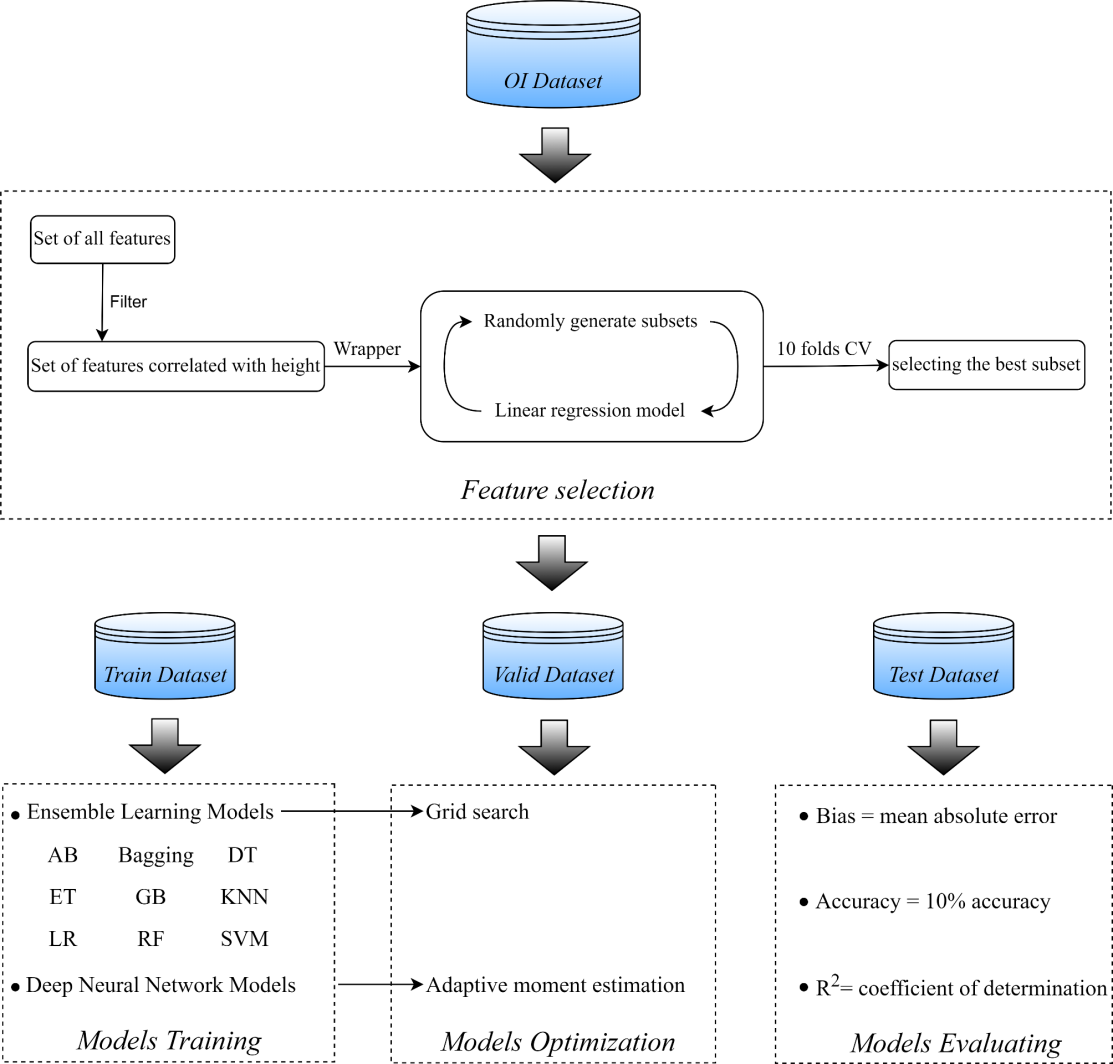
The main steps of the predictive system. Filter is the method of identifying the features correlated with height. Wrapper is the method of selecting the best subset of features. AB: AdaBoost classifier, Bagging: Bootstrap aggregating, DT: Decision tree, ET: Extra Tree, GB: Gradient boosting classifier, KNN; K-nearest neighbor, LR: Linear Regression, RF: Random forest, SVM: Support vector machine

After the initial selection of features using both filter and wrapper methods, we applied ensemble machine-learning (EML) and deep-learning (DL) models to predict the height of children with Osteogenesis Imperfecta (OI). The EML model integrated various algorithms, including the AdaBoost classifier (AB), Bootstrap aggregating (Bagging), Decision Tree (DT), Extra Tree (ET), Gradient Boosting Classifier (GB), K-nearest Neighbor (KNN), Linear Regression (LR), Random Forest (RF), and Support Vector Machine (SVM). This ensemble approach was subsequently optimized using Grid Search (GS), which facilitated the fine-tuning of parameters to enhance model performance. In parallel, we developed a deep-learning model using the Keras framework within TensorFlow. This deep neural network (DNN) was specifically optimized with the Adaptive Moment Estimation (Adam) technique, aiming to refine the learning process and improve predictive accuracy. The comprehensive methodology encompassed feature selection, model construction, training, optimization, and evaluation. These steps were meticulously designed to ensure robustness and reliability in height prediction for children with OI, as depicted in (Fig. 1).

### OI data set

The cross-sectional data of this study encompassed 323 participants with diagnosis of OI, date extracted for analysis including age at enrollment, gender, Sillence classification, height and weight at enrollment, area bone mineral density (aBMD) at enrollment, history of femoral rodding (yes or no) and history of tibial rodding (yes or no). The data were measured in accordance with a uniform method by a trained orthopedist. Age at enrollment was set at a minimum of 3 years old and a maximum of 24 years old. This study majored in exploring the most common children with only collagen genes mutation, children with other genes related to OI were excluded. Participants were classified into OI types I, III and IV according to clinical features, with genotypic information utilized for further verification when available. Height was measured as vertical distance from vertex to sole of the foot, which was performed by height measuring scale to the nearest 0.1 cm. The supine length was measured for subjects without ability of standing. Weight was measured using digital scale to the nearest 0.1 cm. aBMD was measured at the lumbar spine (L1-L4) using a DPX Bravo device (3030 Ohmeda Dr Madison, Wisconosin USA). Results for height, weight and aBMD at enrollment were transformed into age and gender-matched Z-scores according to national growth reference data [17]. The characteristics of participants included in the study were detailed in (Table 1). All data collected from Children’s Hospital of Soochow University were sorted and managed by Tianjin Medical University General Hospital from 2010 to 2021. The study was respectively approved by Medical Ethical Committee of participating hospital.

**Table 1:** Characteristics of the study population

	Type I	Type III	Type IV
Gender (female/male)	107(44/63)	61(31/30)	155(58/97)
Age (years)	8.00(3.00 ~ 24.00)	12.00 (3.00 ~ 24.00)	9.00(3.00 ~ 23.00)
Height (cm)	124.0(91.0 ~ 180.0)	120.0(71.0 ~ 159.0)	127.0(86.0 ~ 173.0)
Height Z-score	-0.62(-5.93 ~ 3.24)	-3.97(-10.44~-0.02)	-1.64(-6.67 ~ 2.44)
Weight (kg)	25.5(14.0 ~ 90.0)	28.0(8.5 ~ 70.0)	28.0(10.0 ~ 71.0)
Weight Z-score	0.06(-2.79 ~ 5.71)	-2.32(-5.81 ~ 2.67)	-0.71(-5.06 ~ 8.67)
aBMD (g/cm^3^)	0.55(0.26 ~ 1.02)	0.39(0.18 ~ 0.86)	0.44(0.19 ~ 0.99)
aBMD Z-score	-0.70(-4.70 ~ 3.50)	-4.20(-8.30 ~ 1.30)	-2.50(-8.20 ~ 1.20)
History of femoral rodding(Yes/No)	107(32/75)	61(37/24)	155(72/83)
History of tibial rodding(Yes/No)	107(10/97)	61(19/42)	155(30/125)

The values are expressed as median (interquartile range, IQR)

### Features selection: a critical step in predictive models

Features selection is a prerequisite process of building an effective predictive model. After features extraction, our dataset comprised 3 continuous variables (including age, aBMD Z-score, weight Z-score) and 4 discrete variables (including gender, Sillence types, history of femoral rodding and history of tibial rodding). According to previous studies, there is no single feature selection algorithm is universally optimalis universally optimal. We firstly used the filter to remove features significantly uncorrelated with height. Then the wrapper was applied to explore the best subset of features that remained after filtering.

*(i) The filtering process*: The filter is usually applied as a preprocessing step, with the aim of selecting the features correlative with dependent variable. In this study, the correlation analysis was examined by Pearson’s coefficient and spearman’s coefficient for continuous variable and discrete variable respectively. This analysis resulted in the retention of five features, while two were eliminated due to their weak correlation with height (Table 2).

**Table 2:** Correlation between height and features

Features	*P* value
Gender	*p* = 0.001*
Age	*p*<0.001^#^
Sillence types	*p* = 0.007*
Weight Z-score	*p*<0.001^#^
aBMD Z-score	*p* = 0.021^#^
History of femoral rodding	*p* = 0.532*
History of tibial rodding	*p* = 0.596*

* Pearson correlation coefficient (PCCs), ^#^ Analysis of variance (ANOVA)

*(ii) The wrapper method*: The wrapper uses machine learning model to choose best subset of features. Initially, we generated 26 potential subsets (excluding those composed solely of one feature) through random permutations of the five retained features. Subsequently, a linear regression model, enhanced by 10-fold cross-validation (CV), was employed to determine the subset that demonstrated the best performance (Table 3).

**Table 3:** Correlation between height and features

Subset	Features	*R* ^2^	CV score
1	Gender, Age, Sillence types, Weight Z-score, aBMD Z-score	0.773	8.569
2	Gender, Age, Sillence types, Weight Z-score	0.771	8.621
3	Gender, Age, Sillence types, aBMD Z-score	0.699	10.139
4	Gender, Age, Weight Z-score, aBMD Z-score	0.736	8.729
5	Gender, Sillence types, Weight Z-score, aBMD Z-score	0.122	17.236
6	Age, Sillence types, Weight Z-score, aBMD Z-score	0.771	8.676
7	Gender, Age, Sillence types	0.675	10.811
8	Gender, Age, Weight Z-score	0.717	8.709
9	Gender, Age, aBMD Z-score	0.649	10.076
10	Gender, Sillence types, Weight Z-score	0.122	17.169
11	Gender, Sillence types, aBMD Z-score	0.060	17.892
12	Gender, Weight Z-score, aBMD Z-score	0.121	17.202
13	Age, Sillence types, Weight Z-score	0.769	8.779
14	Age, Sillence types, aBMD Z-score	0.690	10.358
15	Age, Weight Z-score, aBMD Z-score	0.729	8.626
16	Sillence types, Weight Z-score, aBMD Z-score	0.100	17.380
17	Gender, Age	0.557	10.827
18	Gender, Sillence types	0.052	17.772
19	Gender, Weight Z-score	0.121	17.123
20	Gender, aBMD Z-score	0.056	17.783
21	Age, Sillence types	0.670	10.948
22	Age, Weight Z-score	0.712	8.766
23	Age, aBMD Z-score	0.631	10.299
24	Sillence types, Weight Z-score	0.099	17.332
25	Sillence types, aBMD Z-score	0.026	18.138
26	Weight Z-score, aBMD Z-score	0.097	17.345

1 $$\:CV(k)=\frac{1}{k}\sum\:_{i=1}^{k}MAEi$$

2 $$MA{E_{(i)}} = 1/n\sum\nolimits_{(i = 1)}^n {|{\text{y}}i - yi|}$$

### Data division

The OI data set was divided into a training set, a validating set and a test set with a ratio of 3:1:2. The training set and validating set were mainly used for models training and optimization, and models evaluation was conducted in testing set.

### Models training and optimization

#### Machine learning model

A single machine learning model might not reach the expected performance without theoretical data. Typically, ensemble machine learning (EML) achieved better capacity of prediction, as the combination of heterogeneous machine learning algorithms could alleviate the overall deviations of single model in different vector directions. In this study, we proposed an EML model by averaging the results of the nine single models, including AdaBoost classifier (AB), Bootstrap aggregating (Bagging), Decision tree (DT), Extra Tree (ET), Gradient boosting classifier (GB), K-nearest neighbor (KNN), Linear Regression (LR), Random forest (RF) and Support vector machine (SVM). Then, the grid search (GS) was employed to explore the optimum hyperparameter through testing each value of parameters.

#### Deep learning model

**Fig. 2:**
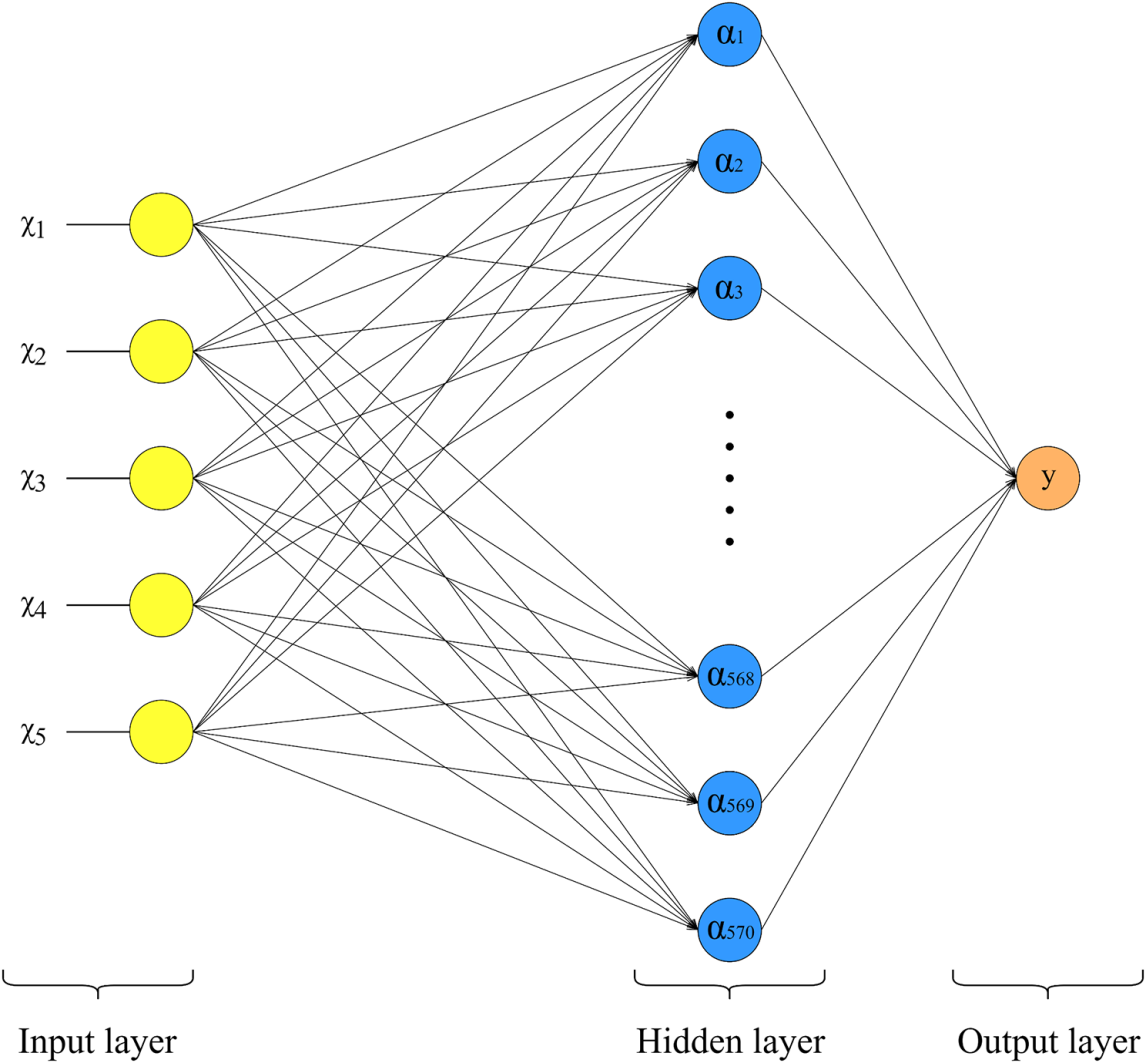
The architecture of the optimized DNN model. χi is the input features, αi is the neuron in hidden layer, y is the output of the model

The optimized DNN model consists of three layers, including input layer, one hidden layer and one output layer (Fig. 2). The input layer, equipped with 5 neurons, was responsible for reading the features in the OI data set, and Standard Scaler method was used for standardization of the data. The hidden layer, containing of 570 neurons, served as classifier that maps samples to each corresponding neuron. This layer utilized a random uniform kernel initializer and relu activation function. The output layer consists of one neuron, formulated as follows:

3 $${Y_i} = {\omega _{\left( {{\rm X}1} \right)\alpha i}}{{\rm X}_1} + {\omega _{\left( {{\rm X}2} \right)\alpha i}}{{\rm X}_2} + \ldots + {\omega _{\left( {{\rm X}j} \right)\alpha i}}{{\rm X}_j} + {\text{ }}b$$

In which y_i_ is the output of the i-th node of the hidden layer, ω_(Χj)αi_ represents the weight of j-th input feature to the i-th neuron of the hidden layer, andΧ_j_ is the j-th input feature, b is the bias.

The Model.fit facilitated the training of the DNN by adjusting the weights of the neurons based on the input data. The adaptive moment estimation (Adam) was employed to optimize the DNN model. It is an easy implementation and efficient computing optimization method based on adaptive lower-order moment estimates. Different from the Stochastic Gradient Descent (SGD), Adam can easily search the optimum hyperparameters for our DNN models by iteratively updating the network weights, in addition, the learning rate of parameters in each iteration is within a certain range, and the overall parameter value is relatively stable. To prevent overfitting, early stopping was implemented to halt training once a predefined performance threshold was achieved.

#### Parameters in DNN

*Hidden Layer.* Since single large hidden layer was adequate for a continuous mapping from one finite space to another, one fully connected layer was selected in this study with relatively small samples.

*Number of hidden layer neurons.* There was no recognized method to determine the number of neurons in the hidden layer. Inappropriate less neurons will reduce the accuracy of the model, nevertheless too many neurons in hidden layer will exhaust computing resource. The number of neurons has adapted from 50 neurons to 700 neurons, and the optimal number of neurons was finally set at 570 with the lowest mean absolute error (MAE) (Table 4).

**Table 4:** The number of neurons for DNN

Number of neurons	Mean absolute error (MAE)	Number of neurons	Mean absolute error (MAE)
20	8.358	570	6.223
180	7.932	571	6.241
280	6.895	572	6.631
480	6.653	573	6.442
560	6.621	574	6.388
565	6.545	575	6.361
566	6.462	580	6.541
567	6.426	600	6.624
568	6.338	680	6.870
569	6.287	880	7.828

*The Number of Iterations.* The DNN model converges when the number of iterations reaches 200 times with the error reaching the goal at 0.001.

### Evaluating models

The performance of the models were assessed using three primary metrics: bias, accuracy, and R^2^. Bias refers to the Mean Absolute Error (MAE), which quantifies the average magnitude of the errors in predictions, irrespective of their direction. Accuracy is defined as the proportion of predictions where the predicted height deviates by no more than 10% from the actual measured height. R^2^, or the coefficient of determination, measures the proportion of variance in the observed data that is predictable from the model inputs, thus indicating the fitting degree of the predictive models.

### Statistical methods

Statistical results were analyzed by using the software SPSS 25 (IBM Corporation, Armonk, New York). Normality of distribution was evaluated using the Shapiro-Wilk tests. Continuous variables were described as median and interquartile ranges, and were compared among the groups using the Kruskal-Wallis test with Bonferroni’s post hoc comparisons for more than two groups because of the asymmetric distribution of data after the normality test. In order to compare the growth patterns in different gender and OI types, data were divided into one-year intervals, with the lowest bin at 3 years and the highest bin at 24 years, to acquire the age at which the curves of each category begin to show statistical differences.

## Results

The characteristics of participants included in the study were depicted in Table 1. Overall, 323 participants (133 females and 190 males) were included, consisting of 107 with OI type I, 61 with OI type III and 155 with OI type IV, and the overall age range was 3–24 years old.

### Height Z-score in OI

**Fig. 3:**
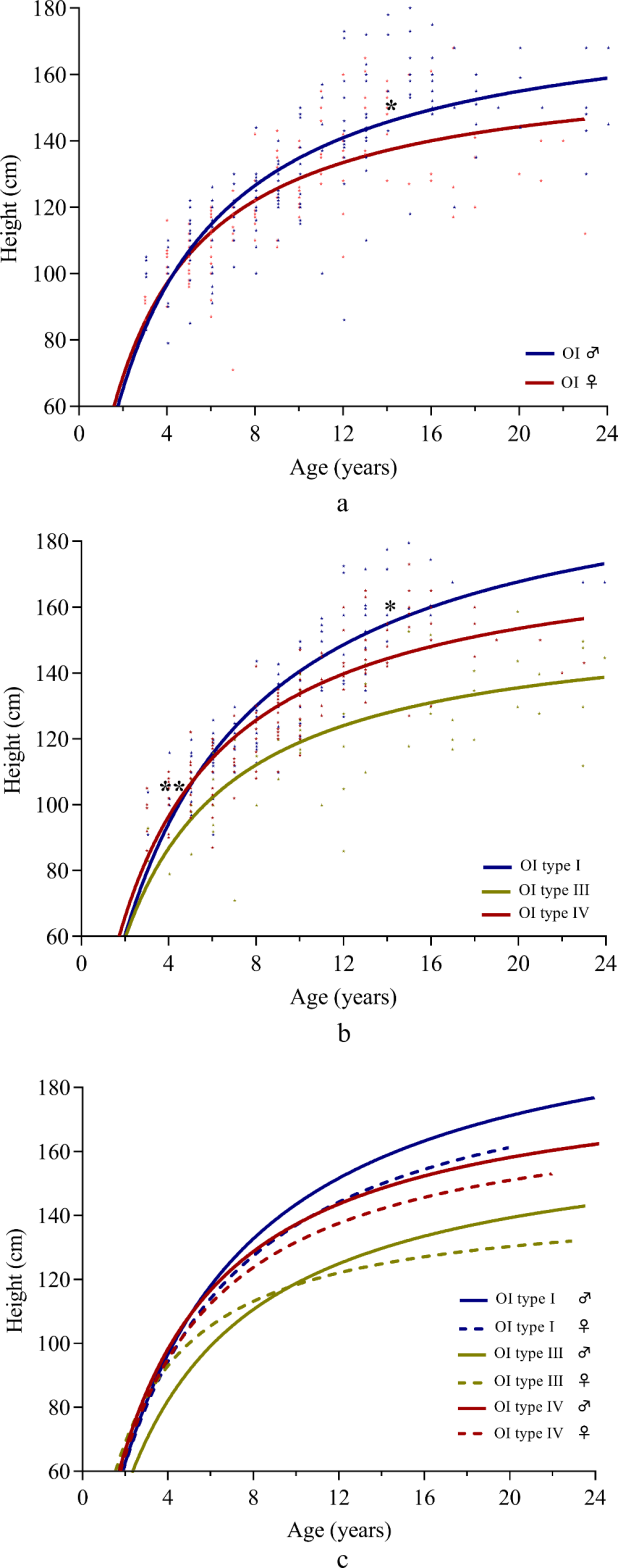
Scatter plot and Fitting curve in OI. The fitting curves applied to estimate growth pattern in different gender and OI types were constructed using the Hyperbola, the function in non-parametrically fitting approaches from Prism 8 software. Data were binned into two groups to construct the fitting curves of height and age, with male and female separated. Data were binned into three groups to construct the fitting curves of height and age, with Sillence I, III and IV separated. * *p* < 0.05, ** *p* < 0.01

The median (interquartile range (IQR)) Z-score of height in OI types I, III and IV respectively were − 0.62 (-5.93 ~ 3.24), -3.97 (-10.44 ~ -0.02) and − 1.64 (-6.67 ~ 2.44) (Table 1). As expected, the overall height of all OI types was short compared with healthy children, participants with OI type III had significantly diminished height compared with OI types I and IV, and individuals with OI type IV had mildly significant decreased height compared with OI type I. The scatter plot and fitting curve were made for comparing height pattern between gender and OI types (Fig. 3). Males with OI are significantly taller than females with OI at the age of 14 (*p* = 0.045), and the gap becomes larger with age increasing. Individuals with OI type III are shorter than those with OI types I and IV at the age of 3 (*p* = 0.006), and the gap increases with age. Individuals with OI type IV are shorter than those with OI type I when they are 10 years old (*p* = 0.028), and the difference increases with age.

### Predictive factors of the height

Usually, optimal predictive factors allow better predictive performance. In this study, we firstly applied the correlation method for feature filtering. Results showing that Gender (*p* = 0.001), age (*p* < 0.001), Sillence types (*p* = 0.007), weight Z-score (*p* < 0.001) and aBMD Z-score (*p* = 0.021) were significantly correlative with height, whereas history of femoral or tibial rodding (*p* = 0.532, *p* = 0.596, respectively) performed low correlation with height. Then, 10-folds cross-validation was used to select the best subset of features, demonstrating that the subset of gender, age, Sillence types, weight Z-score and aBMD Z-score was the final predictive factors of height with lowest CV score at 8.569.

### Performance of EML and DNN models of height prediction

After feature selection, all subjects were randomly divided into training set, valid set and test set with a ratio of 3:1:2. To diminish the overfitting, 10 folds cross-validation (CV) was used to compare the performance of every model, in which data were divided into k subsets so that training model with k-1 subjects and testing models on the remainder repeatedly.

**Fig. 4:**
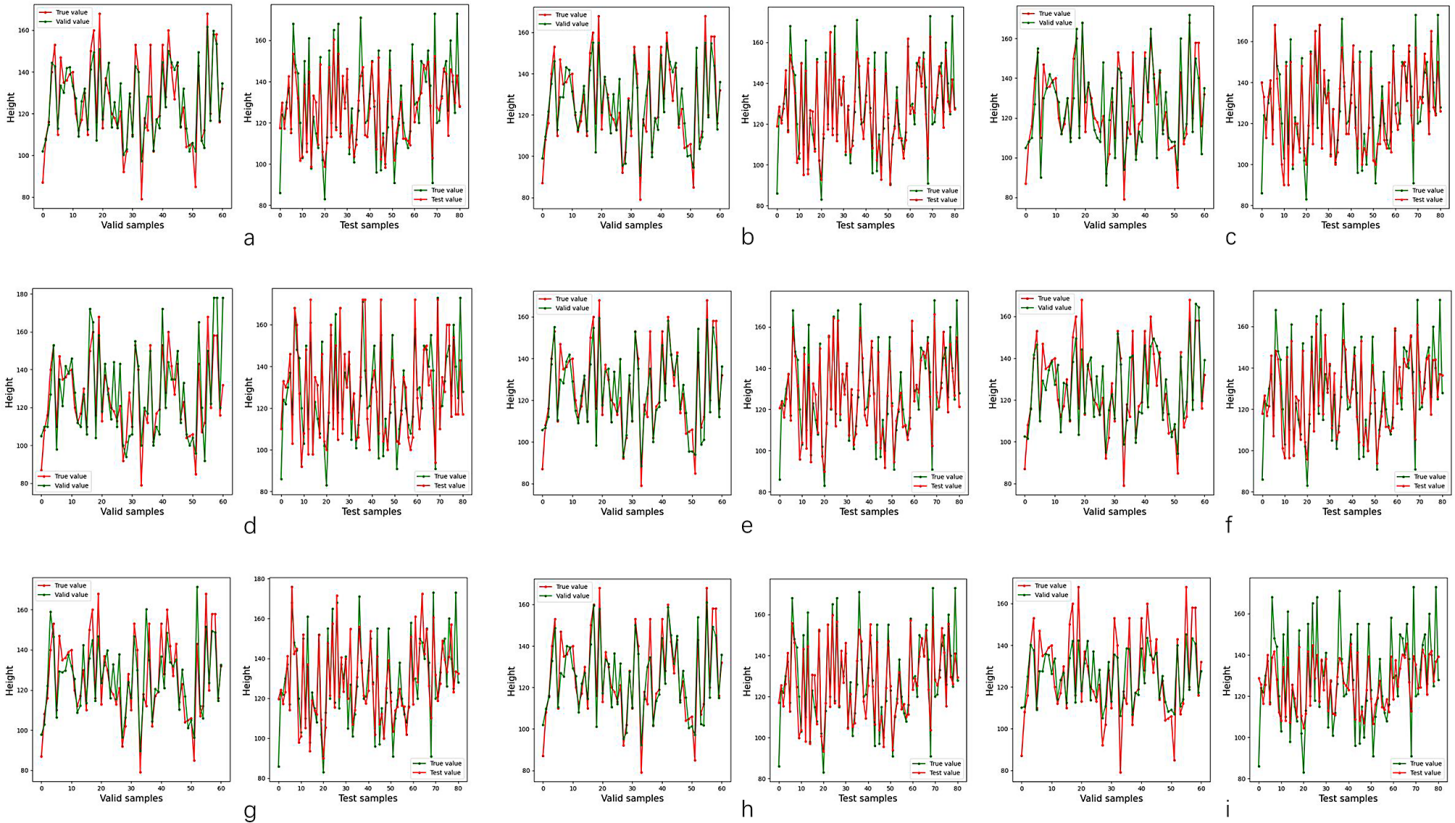
The fitting curve of machine learning models. The fitting curve of each machine model were shown, including (**a**) AdaBoost classifier. (**b**) Bootstrap aggregating. (**c**) Decision tree (**d**) Extra Tree. (**e**) Gradient boosting classifier. (**f**) K-nearest neighbor. (**g**) Linear Regression. (**h**) Random forest. (**i**) Support vector machine. The red lines represent true value and green lines represent predictive value. The left image represents the valid dataset and right image represent the test dataset

**Fig. 5:**
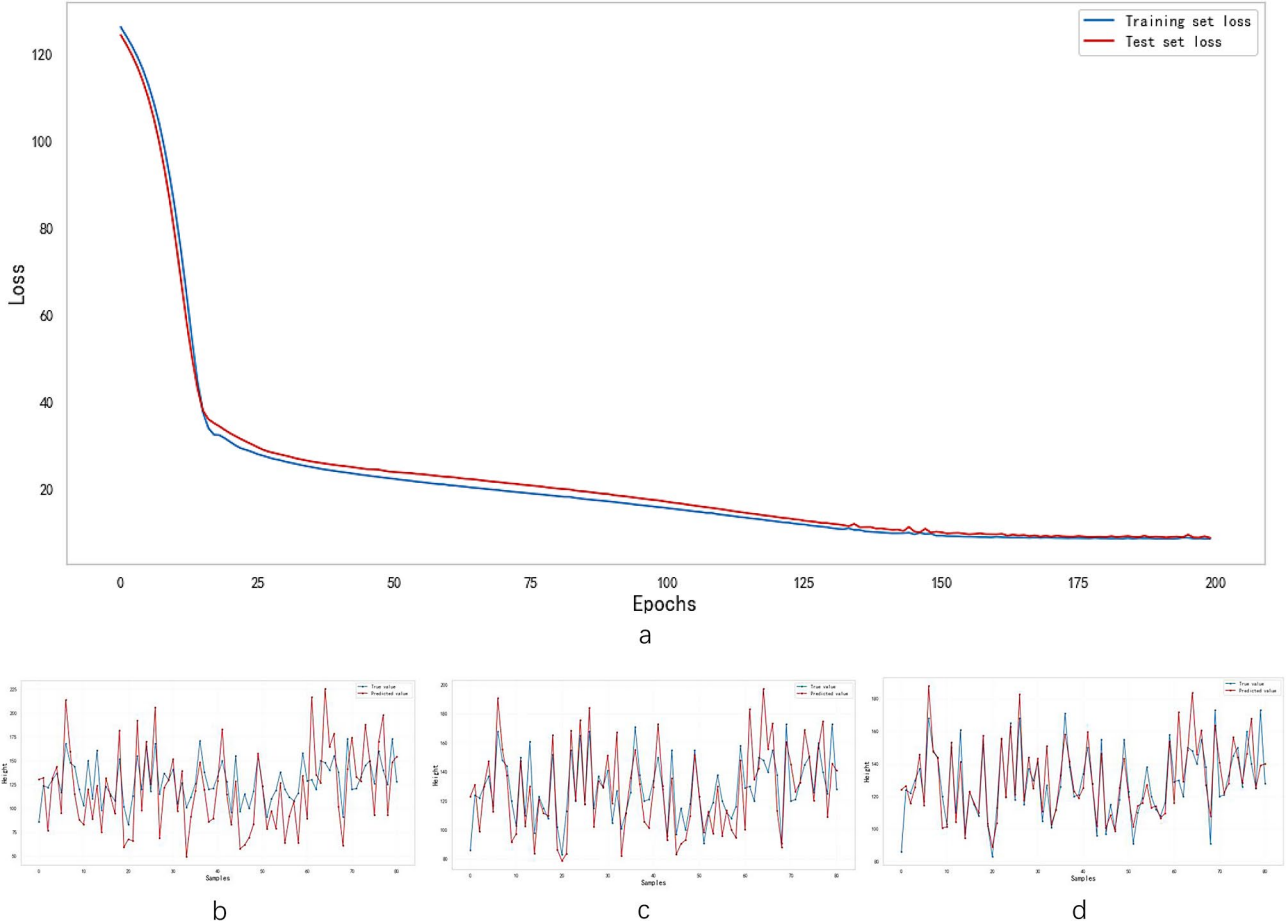
The loss and fitting curve of optimized learning model. (**a**) The loss curve of DNN model, the loss value decreases with iteration increasing, and the DNN model converges at 200 iterations. The fitting curve of DNN model at 40 (**b**), 120 (**c**) and 200 (**d**) iterations, respectively. Red line represents predictive value and blue line represents true value

**Fig. 6:**
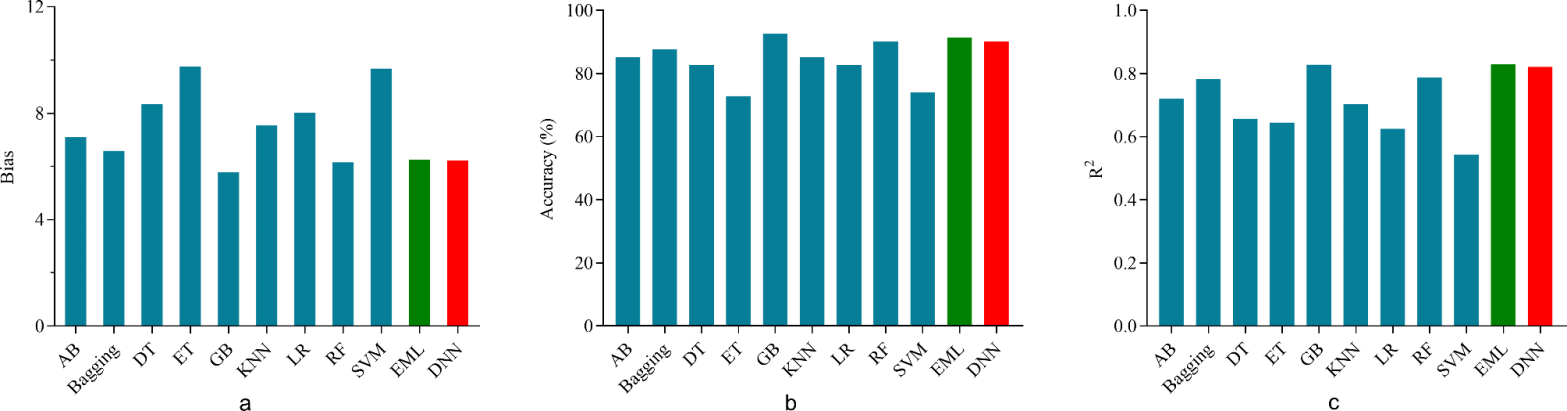
The performance comparison of models. The performance of each model is compared according to bias (**a**), accuracy (**b**) and R^2^ (**c**)

The results of performance of models were shown in (Table 5). The fitting curve of ML and DNN were shown in (Figs. 4 and 5, respectively). In single machine learning, the highest performance is registered by GB and RF (bias = 5.783, accuracy = 92.59%, R^2^ = 0.828) and (bias = 6.155, accuracy = 90.12%, R^2^ = 0.788), respectively. The performance of the ensemble machine learning (bias = 6.250, accuracy = 91.36%, R^2^ = 0.825) was better than single machine learning (*p*< 0.05) except for GB and RF. The DNN have reached the same optimal performance (bias = 6.223, accuracy = 90.12%, R^2^ = 0.821) compared with GB, RF and EML (*p*> 0.05) (Fig. 6).

**Table 5:** The performance comparison of models

Model		Bias	Accuracy	*R* ^2^
Single machine learning	AB	7.100	85.19%	0.720
	Bagging	6.588	87.65%	0.783
	DT	8.346	82.72%	0.657
	ET	9.753	72.84%	0.645
	GB	5.783	92.59%	0.828
	KNN	7.538	85.19%	0.704
	LR	8.025	82.72%	0.625
	RF	6.155	90.12%	0.788
	SVM	9.676	74.07%	0.543
Ensemble machine learning		6.250	91.36%	0.825
Deep neural networks		6.223	90.12%	0.821

AB: AdaBoost classifier, Bagging: Bootstrap aggregating, DT: Decision tree, ET: Extra Tree, GB: Gradient boosting classifier, KNN; K-nearest neighbor, LR: Linear Regression, RF: Random forest, SVM: Support vector machine

## Discussion

It is well known that impaired linear growth is salient hallmark of individuals with OI, but there is a lack of systematic studies on height parameters in OI. In this study, through reporting a large cross-sectional data of individuals with OI in China, we identified the height parameters in different gender and OI types, and ultimately integrated a detailed assessment of these data into clinical management. Moreover, we identified key predictive factors for height and developed multiple machine learning models to predict height variations in OI, enhancing the practicality of monitoring growth and facilitating timely interventions.

As expected, the negative effect of OI on height was found in our cohort, and the mostly affected crowd was OI type III, followed by OI types IV and I. This aligns with earlier studies, Lund et al. first reported height Z-score of OI are compromised compared with unaffected relatives in 1999 [8]. Similarly, Vetter et al. documented the height and weight of type III patients were statistically lower than those with OI types I and IV in a cohort including 127 children [18]. Besides, our study found the significant gap of height between gender in OI was emerged at the age of 14, and the significant difference of height between OI types was found at the age of 3 (OI type III vs. OI types I and IV) and at the age of 10 (OI type I vs. OI type IV).

The predictive factors for height of individuals with OI was not completely clarified. Lauren et al. compiled longitudinal data from 100 children and found that gender and OI subtypes, rather than mutated collagen genes, significantly affected the height of individuals with OI [19]. In their studies, male are taller than female at all ages, and individuals with OI type III are shorter than those with OI types I and IV. Although several studies reported that being overweight is common feature in severe OI types [20–22], the literature is sparse on the correlation between weight and height in OI. The significant correlation between weight and height was found in our study. Bisphosphonate therapy is recognized as standard-of-care for individuals with OI. Zeitlin et al. observed significant height gain in response to 4-year of pamidronate treatment in children with moderate to severe OI [23]. Similarly, studies concluded that other cyclical anti-osteoporosis therapy also increased height Z-score in children with OI [24, 25]. But recent meta-analyses implied no significant effect of bisphosphonate therapy on height [26]. In our cohort, 90% received cyclical anti-osteoporosis therapy, and the various anti-osteoporosis therapy was given at different ages and over different periods of time, thereby limiting our aims of exploring the effect on height. Instead, we found aBMD Z-score was highly correlated with height of OI. Collectively, we attempt to construct predictive models of height using these factors significantly correlated with height, including gender, age, Sillence types, weight Z-score and aBMD Z-score.

Machine learning (ML) offers a robust framework for deriving insights from data features without explicit programming. ML models coupled with medical data can guide all medical administrators, medical staff, patients and their families. ML has great potential as a supplementary source of medical information in guiding the process of diagnosis and medical decision making. After identifying the significant predictive factors of height, multiple machine learning models were established. Precise prediction of the height of OI allows guardians and physicians to timely monitor the growth parameters so that the personalized intervention schedule can be made for the individual patient. A single machine learning model might not reach the expected performance without theoretical data. Ensemble machine learning, combining several single mathematic models, was proposed for improving precision of predictive results, as the bias of single models in different directions would be balanced. Xun Li et al. completed prediction of glomerular fltration rate (GFR) by ensemble learning with high precision [15]. Besides, Yang, J J et al. demonstrated that the ensemble learning outperformed the single models in cataract detection tasks[28]. In our study, the performance of the ensemble machine learning was better than single models except for GB and RF. Further, we believe the precision of ensemble machine learning can be improved with using senior combination than average. Recently, the deep neural network has made big success in medical setting, but the advantages of DNN were handling complex data with many covariates, such as image, text, and speech recognition. As for tabular data and simple regression in our study, the DNN performance no better outcomes for height prediction. Consequently, after balancing the precision of predictive model and computing resource, the single models (GB and RF) and ensemble learning model can be efficient on height prediction for individuals with OI. Despite these advancements, our study has limitations, primarily due to its retrospective nature and the representation of only Chinese patients with OI. The inability to capture comprehensive clinical data may have led to the omission of other relevant height-related factors. Moving forward, a multi-center clinical study would help to generalize these findings and overcome the current study’s limitations.

## Conclusion

In this study, we offered linear growth patterns in different gender and OI types, which can be helpful in making disorder-specific growth curve. Then, several significant predictors of height were identified and multiple predictive models were proposed. The performance of predictive models was compared by MAE, Accuracy and R^2^, which is straightforward to physicians than other believe valuation metrics. Based on our study, we believe the models we proposed could contribute to timely monitoring of growth and early intervention in setting of OI.

## Data Availability

The datasets cannot be openly shared due to the need to protect study participant privacy, such as name and results of gene test. The datasets used and analysed during the current study can only be provided upon reasonable request to the corresponding author.
